# Diurnal fuel moisture content variations of live and dead *Calluna* vegetation in a temperate peatland

**DOI:** 10.1038/s41598-024-55322-z

**Published:** 2024-02-27

**Authors:** C. H. M. Lewis, Kerryn Little, Laura J. Graham, Nicholas Kettridge, Katy Ivison

**Affiliations:** 1 School of Geography, Earth and Environmental Sciences, University of Birmingham, Birmingham, UK; 2 Biodiversity, Ecology and Conservation Group, International Institute for Applied Systems Analysis, Vienna, Austria

**Keywords:** Environmental sciences, Hydrology, Ecophysiology

## Abstract

The increasing frequency and severity of UK wildfires, attributed in part to the effects of climate change, highlights the critical role of fuel moisture content (FMC) of live and dead vegetation in shaping wildfire behaviour. However, current models used to assess wildfire danger do not perform well in shrub-type fuels such as *Calluna vulgaris,* requiring in part an improved understanding of fuel moisture dynamics on diurnal and seasonal scales. To this end, 554 samples of upper live *Calluna* canopy, live *Calluna* stems, upper dead *Calluna* canopy, dead *Calluna* stems, moss, litter and organic layer (top 5 cm of organic material above mineral soil) were sampled hourly between 10:00 and 18:00 on seven days from March-August. Using a novel statistical method for investigating diurnal patterns, we found distinctive diurnal and seasonal trends in FMC for all fuel layers. Notably, significant diurnal patterns were evident in dead *Calluna* across nearly all sampled months, while diurnal trends in live *Calluna* canopy were pronounced in March, June, and August, coinciding with the peak occurrence of UK wildfires. In addition, the moisture content of moss and litter was found to fluctuate above and below their relative ignition thresholds throughout the day on some sampling days. These findings underscore the impact of diurnal FMC variations on wildfire danger during early spring and late summer in *Calluna* dominated peatlands and the need to consider such fluctuations in management and fire suppression strategies.

## Introduction

Heathlands and peatlands are found within temperate regions^1^ and represent globally important carbon stores^2^. Heathlands are upland or lowland habitats with acidic soils or shallow peat layers^1^ and peatlands contain peat soils and blanket bogs. Both ecosystems are notably dominated by *Calluna vulgaris* (hereon *Calluna*), a dwarf shrub-type heather. Other key vegetation species include purple moor grass (*Molinia caerulea*)^3^. These habitats are under threat from the increasing frequency and severity of wildfires^3,4^. Deposits of ash, fine sediment and organic materials also have the potential to pollute waterways and accumulate in the soil substrate^5^. By 2050, it is predicted that the average UK summer temperature will rise by 2.5 °C while rainfall will decrease by 16%, ultimately lowering the fuel moisture content (FMC) of fuels^6,7^ and increasing wildfire occurrence^8^.

The FMC of different *Calluna* fuel layers is an important factor controlling fire behaviour^9^. Low FMC in the *Calluna* canopy can result in faster rates of fire spread, and low FMC in the moss/litter layers can result in more severe fires^10^ leading to more consumption of organic matter^11^ and greater release of carbon stores. Early spring is one of the major periods of high fire risk in such habitats^12^. During the spring, weather conditions in upland areas can be highly variable and fluctuate below and above freezing. Cold freezing conditions and dry warm conditions both lead to low FMC and hence higher wildfire danger. Physiological drought can also impact the ability of *Calluna* to retain moisture; for example, frost and wind can damage the cuticles of the leaves preventing their ability to control evapotranspiration, and frozen ground can prevent root uptake of moisture, leading to below-average FMC^13,14^.

Previous studies have focused on improving the ability to predict FMC of live and dead *Calluna* vegetation layers and the associated fire risk by defining FMC thresholds for which materials can ignite. Davies and Legg^9^ characterised FMC thresholds in *Calluna* using ignition tests along FMC gradients. When the FMC in the lower canopy of *Calluna* was above 70% ignition tests failed, whereas at 60% fires would ignite and develop rapidly. Santana and Marrs^15^ developed a predictive model of the ignition of *Calluna* and *Sphagnum* moss in peat-dominated heathlands as a function of FMC and found that the FMC and proportion of dead materials within the *Calluna* vegetation influence the probability of ignition.

It is now well-known that during the transition from spring into summer, *Calluna* undergoes a “greening up” stage, where its leaf green area increases and hence so does the FMC before the summer drying starts^16^. However, there are very few studies investigating diurnal trends of FMC in *Calluna* and how these patterns change on a seasonal basis^17,18^.

Diurnal patterns in fire behaviour were first characterised by Beall in 1934^19^, with further investigation into the associated diurnal patterns in FMC carried out by Van Wagner in 1977^20^. More recent studies on diurnal FMC have typically been carried out in American forests and grassland environments^21,22^, showing significant diurnal patterns in moisture content. Banwell et al.^21^ found all forest floor litter components experienced at least a 6% change in FMC during the day, and an 11% change in FMC was the greatest range they recorded. Similarly, Livingston and Varner^22^ found a 4–12% variation in FMC of grassland throughout the day. This is significant considering the work by Davies and Legg^3^ which documented a 10% change in FMC in live *Calluna* signifies the difference between fires developing rapidly and failing to ignite fires. Davies^18^ investigated diurnal FMC from July–August but did not sample the early spring period when upland heathland and peatland fires are most frequent. There is a clear need to characterise the diurnal variation of FMC in live and dead *Calluna* vegetation and the underlying moss, litter and organic soil layers across the entire fire season so they can be compared to pre-defined ignition thresholds, as significant diurnal patterns could cross ignition thresholds. Diurnal patterns in FMC could also affect fire behaviour such as rate of spread. As the FMC of *Calluna* changes on a seasonal scale^14^, the diurnal pattern may also change and could be significant from a management and fire suppression perspective.

Here we examine the diurnal variation of FMC of live and dead fuel loads within a *Calluna vulgaris* dominated heathland and determine how the diurnal pattern in FMC varies on a seasonal basis. We also investigate whether the range in FMC throughout each sample day is linked to the range in weather (temperature and humidity) to assess the potential drivers of this diurnal variation. We expect the daily FMC measures to be higher at the earliest and latest measurement times, following a positive quadratic curve.

## Methods

### Site description

**Figure 1:**
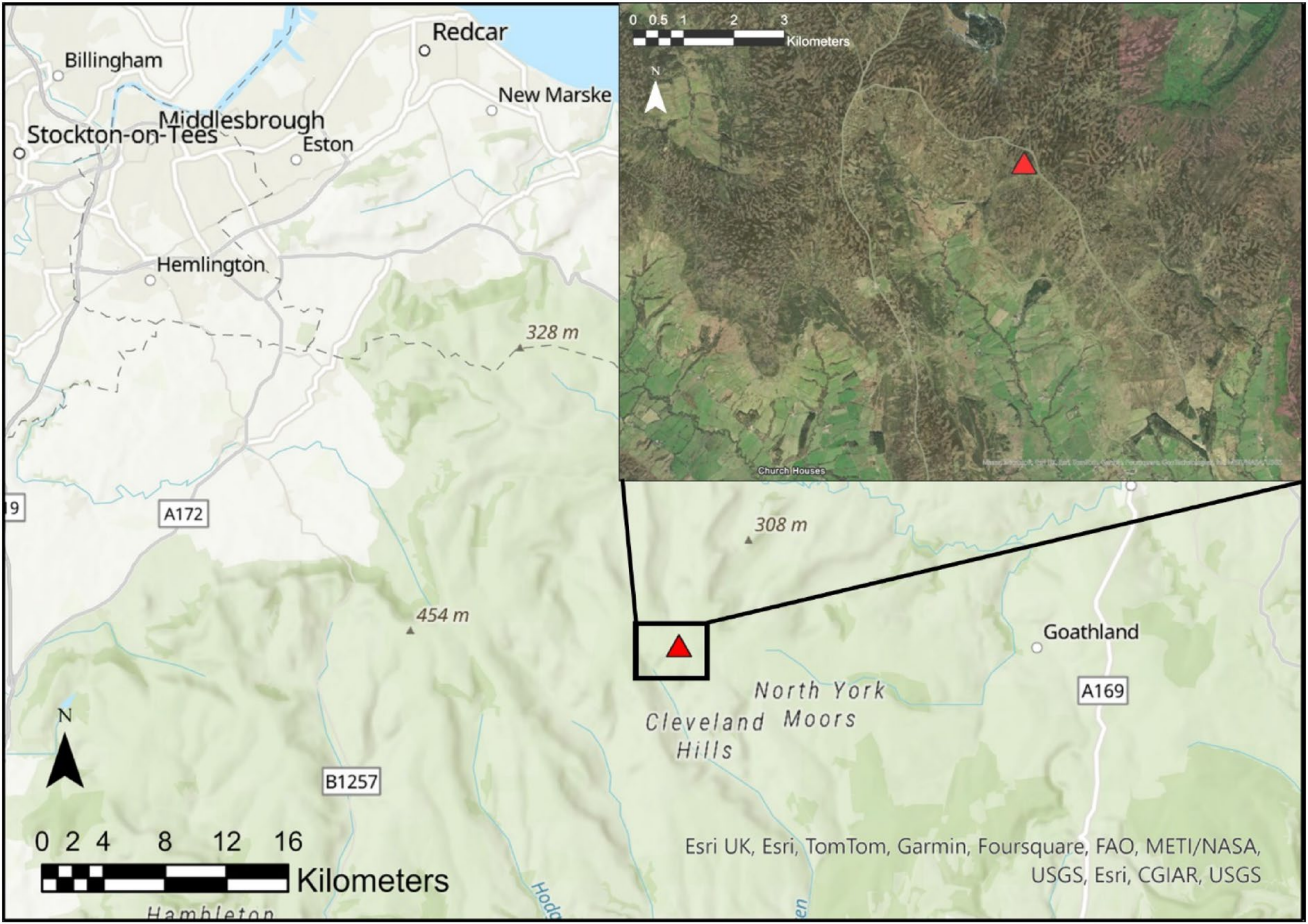
Location of the sample site in the estate, North York Moors. Main map sources: Esri, Airbus DS, USGS, NGA, NASA, CGIAR, N Robinson, NCEAS, NLS, OS, NMA, Geodatastyrelsen, Rijkswaterstaat, GSA, Geoland, FEMA, Intermap and the GIS user community. Satellite inset map source: Esri, Maxar, Earthstar Geographics, and the GIS User Community. Created using ArcGIS Pro v 3.0.2 © 2022 Esri Inc.

The North York Moors national park is an upland area in North-Eastern Yorkshire that contains some of the largest expanses of heather moorland in the UK. The habitat has been managed for centuries by rotational burning to create a mosaic of mixed-age heathland to improve grouse habitat and prevent the build-up of fuel (Fig. 1). The area chosen for this study is located on an estate in the Cleveland Hills (54° 24′ 03.4″ N 0° 55′ 44.6″ W). At an elevation of 408 m above sea level, the site is located at the top of a valley with a slope of 3.8° in a southwest direction. The site is classified as a blanket peat bog with wet heather moor^23^, where *Calluna vulgaris* is the dominant heather species.

### Sampling procedure

**Figure 2:**
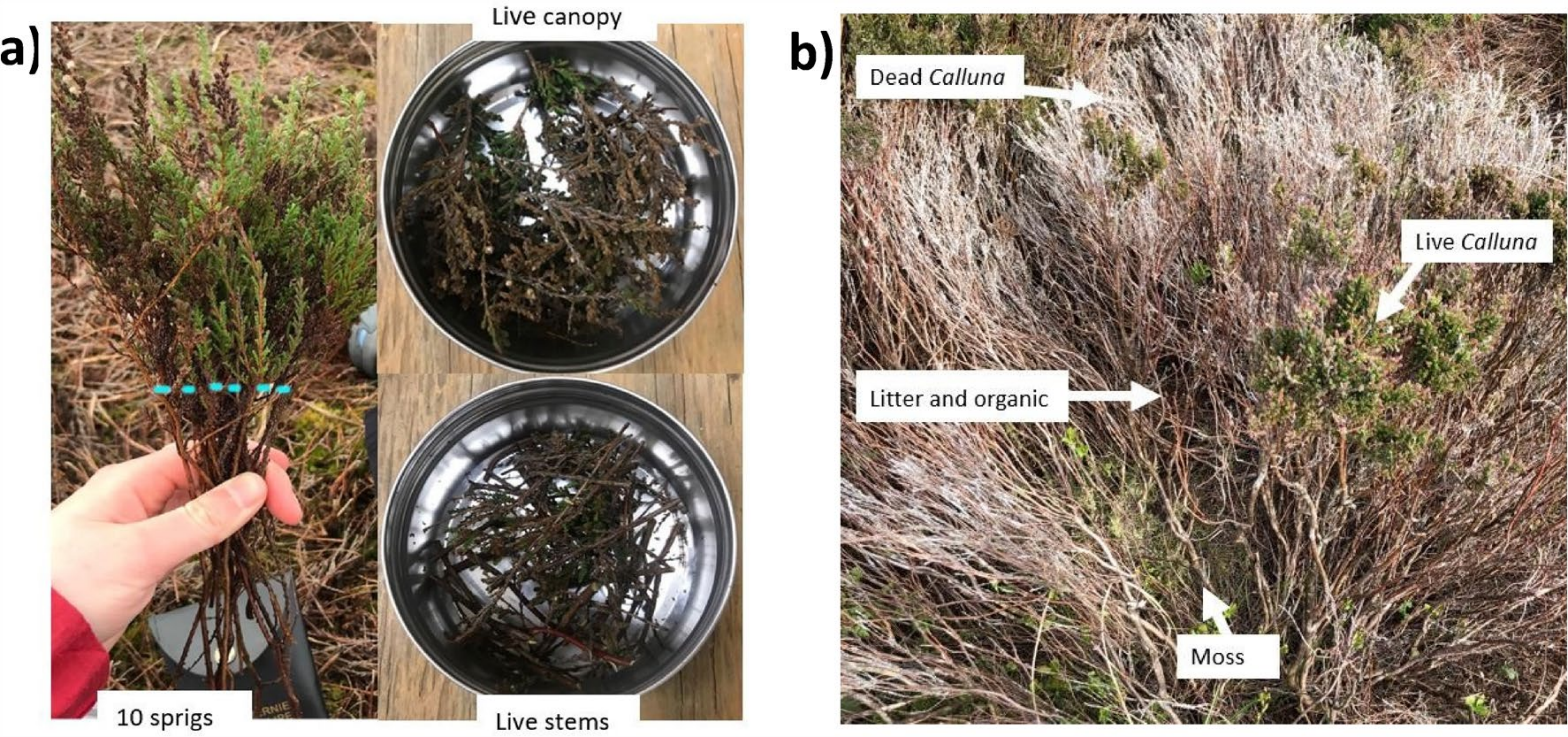
(**a**) Example of how the live *Calluna* sprigs were separated into the upper live canopy and live stems, the dotted line representing the dividing line, (**b**) Sample area containing all vegetation layers.

Sampling was undertaken on the following seven days in 2022: 19th, 20th and 26th March; 21st April; 14th May; 15th June; 6th August. Extra samples were taken in March as this is a key fire period in these ecosystems in the UK. Herein, sample days during April–August will be referred to by the month of sampling only. Individual sampling days were selected to avoid wet antecedent conditions and to maximize diurnal humidity variations. In total, 554 samples were collected and analysed. On each day of sampling the vegetation was sampled on the hour every hour from 10:00 to 18:00 as this is the period of highest fire risk during the day and will help to inform sampling strategies. On most days only one sample was taken per hour, but on March 19th and March 26th two samples were taken per fuel by two separate individuals. Seven different vegetation layers were sampled (Fig. 2): upper live canopy, live stems, upper dead canopy, dead stems, moss, litter and organic layer (top 5 cm of organic material above mineral soil).

The sampling procedure was adapted from Norum and Miller^24^ and Little et al.^25^. Ten random sprigs of live and dead heather were collected haphazardly along a 20 m transect covering a representative area of the site. Each sprig was approximately 10–20 cm long and was taken vertically through the centre of the shrub to avoid the outer sprigs of heather that are exposed and possibly damaged by grazing sheep. The sprigs were then cut into one-inch segments, separating the top canopy and lower stems (Fig. 2a). Moss, litter and organic layers samples were collected haphazardly from five locations along the same 20 m transect. The moss was pulled from the ground and any brown and decomposing sections of moss beneath were removed from the sample. The litter was collected from the base of *Calluna* shrubs, ensuring not to collect any decomposing litter that may be considered the start of the organic layer. Finally, the organic material was collected after sweeping away the litter layer, and a 5 cm deep core of the organic layer was removed. All samples from each fuel layer were combined and placed in a metal tin container and taped to prevent moisture loss. Temperature and humidity measurements at the times of sampling were taken from the Blakey Ridge weather station^26^ which is located roughly 1.8 miles (2.8 km) from the sample site.

FMC is a measure of the amount of water in the fuel layer and is expressed as a percentage of the dry weight of that specific fuel (gravimetric moisture content). To calculate the FMC from the samples collected, the tape was removed from the tins which were then weighed to obtain the wet weight. The samples were placed in a dehydrator set to 80 °C for 48 h and then weighed again to obtain the dry weight. The FMC for each sample was then equal to:

$${\text{FMC}} = \left( {{\text{W}} - {\text{D}}} \right)/\left( {{\text{D}} - {\text{T}}} \right) \times {1}00$$

where W = wet weight, D = dry weight and T = the weight of the sample tin.

### Data analysis

Due to the expected relationship between time of day and FMC, we fit linear regression models with a quadratic term, which we would expect to be positive. This statistical method has not been used to investigate diurnal patterns before. FMC was the response variable, and time of day the independent variable. We fit a single model for each vegetation layer and for each month of sampling, resulting in 49 models. Model residuals from the models were checked using the package ‘DHARMa’^27^, and they did not deviate from their expected distribution for any model (Supplementary Figure S1). Correlation analysis was then carried out to test whether there was a relationship between the diurnal range in FMC and the diurnal range of temperature and humidity. We used the Shapiro test to check the normality of data distribution, and used Pearson’s correlation for normally distributed data and Spearman’s correlation for non-normally distributed data. All data analysis was undertaken using R version 4.3.0^28^.

## Results

**Figure 3:**
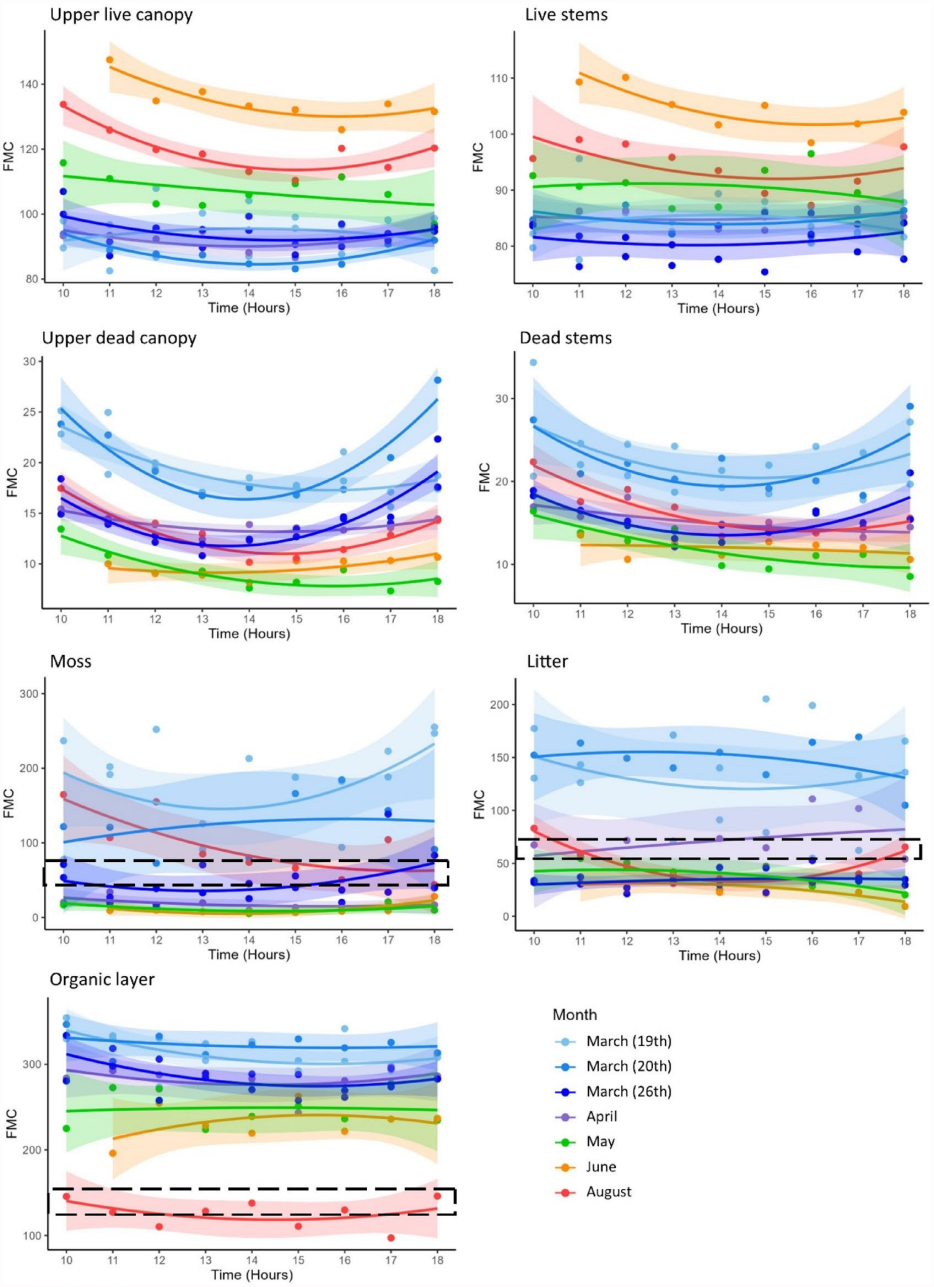
Diurnal and seasonal trends of FMC in the upper live canopy, live stems, upper dead canopy, dead stems, moss, litter and organic layers. The black dashed box represents defined ignition thresholds of the material as reported by Santana and Mars^15^ and Grau-Andrés et al.^29^. The lower and upper bounds of the black dashed box are the M50 (where 50% of ignition tests were successful) and the Mmax (the maximum FMC where ignition occurred). Moss thresholds = 57–80%, litter = 54–61%, organic layer = 125–150%. Ignition thresholds are not shown for live *Calluna* as recorded ignition thresholds (60–70%)^9^ are not within the axis range; all FMC are above threshold. Dead *Calluna* ignition thresholds are not known.

Diurnal variations in fuel moisture were observed for all seven vegetation layers in at least one month, with the majority of models showing a parabola-shaped curve (indicated by a positive quadratic sign; Table 1). Significant diurnal patterns were observed more frequently for dead *Calluna* than live *Calluna* (Table 1, shaded boxes)*.* The moss and litter layers only showed diurnal trends towards the end of the study period, and the organic layer only had significant diurnal patterns on one sample day (March 26th; Table 1). For full summaries of diurnal models see Supplementary Table S1. Dead *Calluna* and surface materials (litter, moss and the organic layer) showed the largest diurnal range in FMC in March, whereas the diurnal range in FMC for live *Calluna* was more consistent across all sample days (Table 2; Fig. 3). Diurnal fluctuations in FMC of surface materials meant that on some sample days FMC dropped below the ignition thresholds only at certain times of day (Fig. 3).

**Table 1:** Regression models with a quadratic term investigating whether FMC diurnal patterns follow parabolic curve for each sample day and at each canopy layer.

		ULC	UDC	LS	DS	L	M	O
March 19th	R^2^	0.04	***0.61***	0.06	*0.26*	*0.05*	*0.20*	*0.31*
	P-value	0.75	*** < 0.01***	0.63	*0.12*	*0.72*	*0.20*	*0.06*
March 20th	R^2^	***0.85***	***0.88***	*0.19*	*0.52*	0.18	0.08	*0.10*
	P-value	*** < 0.01***	*** < 0.01***	*0.52*	*0.11*	0.56	0.80	*0.73*
March 26th	R^2^	*0.24*	***0.77***	*0.05*	***0.63***	0.06	*0.17*	***0.37***
	P-value	*0.13*	*** < 0.01***	*0.70*	*** < 0.01***	0.47	*0.26*	***0.03***
April	R^2^	*0.41*	***0.71***	*0.07*	*0.55*	0.14	*0.33*	*0.16*
	P-value	*0.20*	***0.02***	*0.84*	*0.09*	0.65	*0.30*	*0.60*
May	R^2^	*0.29*	***0.82***	0.09	***0.76***	0.43	*0.39*	< 0.01
	P-value	*0.35*	***0.01***	0.76	***0.01***	0.19	*0.23*	0.99
June	R^2^	***0.76***	*0.54*	***0.71***	0.12	0.50	***0.72***	0.20
	P-value	***0.03***	*0.15*	***0.05***	0.73	0.18	***0.04***	0.57
August	R^2^	***0.86***	***0.90***	*0.38*	***0.86***	***0.86***	***0.65***	*0.19*
	P-value	*** < 0.01***	*** < 0.01***	*0.24*	*** < 0.01***	*** < 0.01***	***0.04***	*0.53*

R^2^ values of above 0.70 indicate good model fit. Bold text indicates that models have significant P-values (< 0.05). Models with a positive quadratic sign (i.e. parabolic curve, expected for diurnal FMC patterns) are shown in italic text and models with a negative quadratic sign (i.e. bell-shaped curve) are in non-italic text. Bold, italic text therefore denotes models with significant positive quadratic signs patterns. ULC = upper live canopy, LS = live stems, UDC = upper dead canopy, DS = dead stems, M = moss, L = litter, O = organic layer. For full model summaries see Supplementary Table S1.

**Table 2:** Range between minimum and maximum FMC (%) observed during each sample day.

Vegetation layer	March 19th	March 20th	March 26th	April	May	June	August
ULC	25	11	20	10	19	22	23
LS	18	6	11	4	12	12	12
UDC	9	11	12	3	6	2	7
DS	17	11	9	5	8	3	9
L	150	64	31	75	34	29	52
M	182	112	122	25	16	21	121
O	81	43	76	54	53	66	49

ULC, upper live canopy; LS, live stems; UDC, upper dead canopy; DS, dead stems; M, moss; L, litter; O, organic layer.

There was no significant correlation between the diurnal range in FMC and the diurnal range in weather variables (temperature or humidity) throughout each sample day. Weakly significant correlation was observed only between the diurnal range in FMC of moss and diurnal range in humidity (0.67; *P* = 0.1; Table 3).

**Table 3:** Correlation analysis between range in FMC per sample day (max FMC—min FMC; Table 2) and range in temperature and humidity on each sample day (Supplementary Table S2) for each fuel layer.

Test		Temperature	Humidity
ULC	Correlation	0.13	0.43
	*P* value	0.77	0.34
LS	Correlation	0.35	0.40
	*P* value	0.44	0.37
UDC	Correlation	0.59	0.45
	*P* value	0.16	0.31
DS	Correlation	0.47	0.63
	*P* value	0.29	0.13
L	Correlation	− 0.18	0.32
	*P* value	0.71	0.50
M	Correlation	0.25	**0.67**
	*P* value	0.59	**0.10**
O	Correlation	− 0.07	− 0.38
	*P* value	0.88	0.40

Weakly significant *P* value (< 0.1) is highlighted in bold.

## Discussion

Dead *Calluna* showed significant diurnal parabola-shaped patterns in almost every month of sampling, whereas for live *Calluna,* significant diurnal patterns were found in March, June and August. This supports similar work by Davies^18^, who found large diurnal variations in the FMC of dead *Calluna* from July–August and shows that diurnal patterns are a significant factor characterizing fuel moisture in dead materials. During the winter and early spring, the relative proportion of dead *Calluna* is at its greatest due to winter die back^13,30^, and the proportion of dead materials may have an important impact on wildfire behaviour, where small changes in the percentage of dead materials can have a disproportionate impact on the severity and spread of wildfires^31^. As a result, during the winter/spring when the proportion of dead materials is highest, the diurnal variability of dead materials' moisture content may be an important factor in wildfire danger. Although ignition thresholds are unknown for dead *Calluna*, low dead FMC increases the risk of fire ignition^14^, which suggests that ignition is more probable in dead *Calluna* when the diurnal FMC is at its lowest.

The upper live canopy had significant diurnal patterns in the months that wildfires are most likely; early spring (March) and mid-late summer in June and August^32^. As live FMC can therefore be impacted by the time of day, FMC predicting models that are calibrated with field samples likely need to take account of diurnal patterns, especially during the early spring and late summer. Significant diurnal trends in early spring could be explained in part by phenological factors such as winter damage to leaves reducing the plants’ ability to regulate water loss, meaning FMCs are correlated with diurnal evaporation^30^. Despite the significant diurnal trends, FMC of all live *Calluna* was above the recorded ignition threshold^9^ which means that diurnal variation is unlikely to affect ignition probability in this study. However, live FMC is linked to wildfire rate of spread^14,29^ which may vary depending on the time of day that a wildfire occurs. Considering diurnal patterns in live FMC is still therefore essential in wildfire management.

Different vegetation layers showed different FMC patterns depending on the time of year. The lowest FMC in the live *Calluna* was in March (Fig. 3), and low springtime FMCs have been recorded by other studies such as Davies et al.^33^. For example, it was noted that the FMC of live C*alluna* decreased during the winter of 2002–2003 from a usual spring value of 80% (similar to levels recorded in this study) to less than 45%^30^. During the winter, freezing conditions and high wind speeds can damage leaf cuticles and cause physiological drought^34,35^. Live FMC can further be lowered by freezing grounds locking up soil moisture and preventing root uptake^14^. However, an alternative explanation for large diurnal ranges and low FMC in March found in this study could be due to an increase in elevated dead fuels from winter browning (plant damage due to frost or wind in early spring^13^) of live materials that were not separated from live *Calluna* sprigs causing large variation between samples. In comparison to the live *Calluna*, the FMC of the dead *Calluna* varied less seasonally, with FMC of ~ 5% at its lowest in May to ~ 30% at its highest in March.

During the early summer when *Calluna* increases its green-leaf area and biomass, the FMC of the upper live canopy increased by over 50% from April–June (Fig. 3). This is a well-described phenomenon^36^ and is consistently shown in FMC studies^16,33^. The diurnal patterns of live *Calluna* were most significant in the late summer months after the growth spurt. However, the fire danger is likely lower during this late summer as FMC remains higher than in spring through the entire diurnal variation.

The rate of wildfire spread is influenced by FMC of the lower ground and moss/litter layers; when the FMC of the moss and litter layers are low enough to ignite, they can substantially increase available fuel loads by 13–67% which can significantly alter fire behaviour^29^. The FMC of the moss and litter layers had their largest diurnal ranges in March, April and August (Fig. 3; Table 2), during which FMC fluctuated above and below the ignition thresholds on some sample days. Diurnal variations in FMC could therefore affect wildfire behaviour by increasing the amount of available fuels and speeding up the rate of spread, since the litter and moss layers can dry to combustible FMCs throughout the day. Models that do not consider the hourly change in FMC will therefore underestimate the wildfire risk. This is highlighted by the fact that for some materials and sample days (March 20th for moss; April for litter), the mean daily FMC is above the ignition thresholds (Supplementary Table S3) but does drop below the threshold at some point during these days. However, on sample days where FMC of moss and litter fluctuated above and below the ignition threshold, diurnal patterns were only statistically significant in August. Despite large diurnal ranges in the moss and litter layers in early spring, the diurnal patterns were not statistically significant and the variation could have been the result of high within-plot variability in FMC.

The influence of surface fuel on wildfire behaviour, from a fuel moisture perspective, is significantly different in late summer compared to springtime as drier conditions lower the organic layer FMC to such an extent that smouldering of the peat is feasible^37–39^. We observed the organic layer moisture content fluctuating on a diurnal scale above and below the ignition thresholds defined by Grau-Andrés et al.^29^. However, for six of the seven sample days there was no significant parabola-shaped pattern of diurnal variation observed, likely the result of high spatial variability in organic FMC^40^. This suggests that diurnal patterns in FMC of the organic layer are not an important factor governing wildfire behaviour and may be more governed by seasonal weather.

We found no strong links between the range of diurnal FMC in each fuel layer and the range in weather variables (temperature or humidity), with the exception of moss where there was a very weak correlation between the diurnal ranges of FMC and humidity. Many studies have found links between weather and FMC: for example, dead *Calluna* is influenced strongly by the weather, likely as it can no longer regulate its moisture content by drawing in water from the ground or respiring as live plants do^41^, and a positive relationship between live FMC and temperature for live *Calluna* and other live materials has also been observed^3^. However, the relationship between diurnal weather patterns and the diurnal range in FMC have not been researched before. More detailed work may therefore be needed to further investigate the potential effects of day and night-time weather, as well as dew and antecedent weather conditions, on observed diurnal fluctuations in live and dead fuel moisture content.

The FMC of vegetation layers is a significant factor governing wildfire behaviour; high FMCs inhibit wildfire risk, and low FMCs increase wildfire danger and ignition probabilities^10^. Improving our understanding of FMC patterns in live and dead materials is crucial for developing an accurate UK fire danger rating system, yet there are no studies characterising diurnal and seasonal patterns of FMC in *Calluna* vegetation layers across the whole fire season. We observed significant diurnal patterns in FMC that varied seasonally. In general, diurnal patterns became more significant during the late summer period and our data suggests there might be significant diurnal patterns in live *Calluna* FMC during the early spring as well. The main implications of this study are that diurnal FMC in live material can impact wildfire danger during the early spring and late summer, which coincides with the two main wildfire seasons, and that diurnal fluctuations in ground materials can result in ignition risk changing throughout the day. From an FMC monitoring perspective, samplers will need to consider the time of day that samples were taken as this can influence measured FMCs and fire danger rating system models should consider the diurnal changes of FMC.

### Supplementary Information

Supplementary Information.

## Data Availability

The datasets generated and analysed in this study are available on Figshare, https://figshare.com/s/66b2b262796ae0133a6b.
