# Supplementary material for: Diurnal fuel moisture content variations of live and dead Calluna vegetation in a temperate peatland
Source: Sci Rep. 2024 Feb 27;14:4815. doi: 10.1038/s41598-024-55322-z (PMC10899638; doi:10.1038/s41598-024-55322-z)
Supplement: Supplementary file 1 — Supplementary Information. [file 41598_2024_55322_MOESM1_ESM.docx]

| Figure S1: Distribution of residuals from regression models with quadratic terms for seven fuel layers across seven sample days from March to August 2022. ULC = upper live canopy, LS = live stems, UDC = upper dead canopy, DS = dead stems, M = moss, L = litter, O = organic layer. | | | | |
| --- | --- | --- | --- | --- |
| Sample day | Residuals plots | | | |
| March 19th | ULC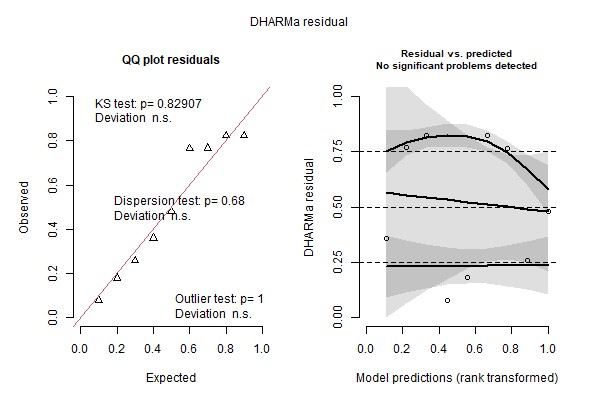 | UDC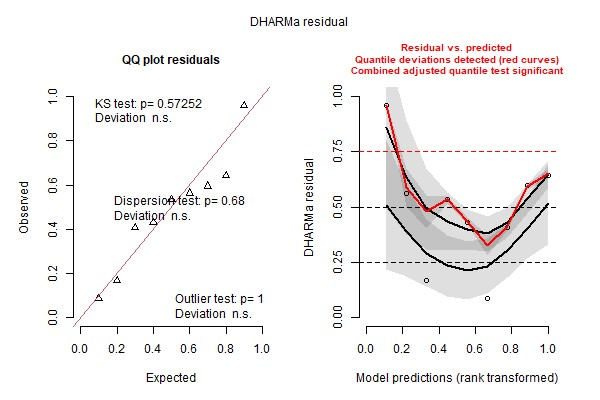 | LS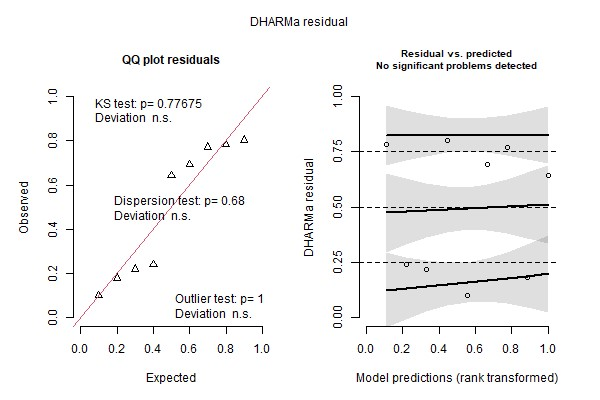 | DS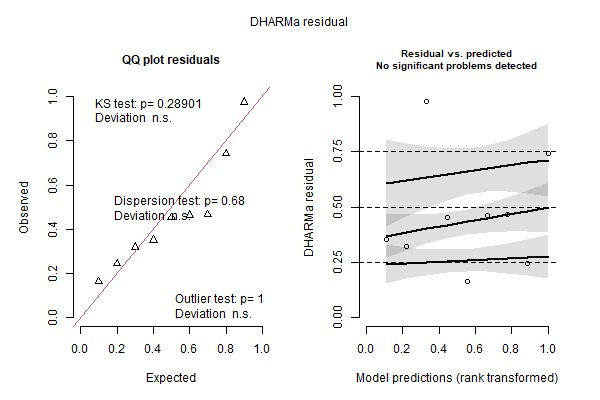 |
|  | M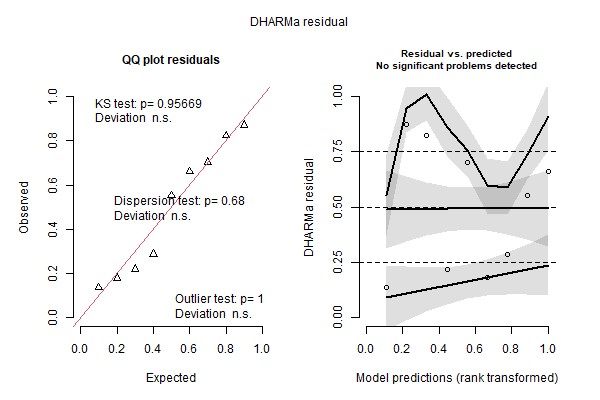 | L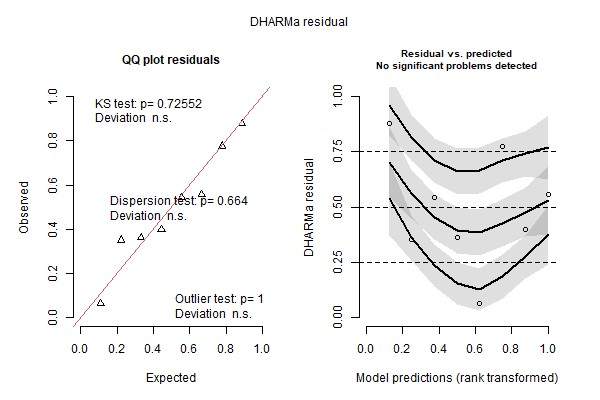 | O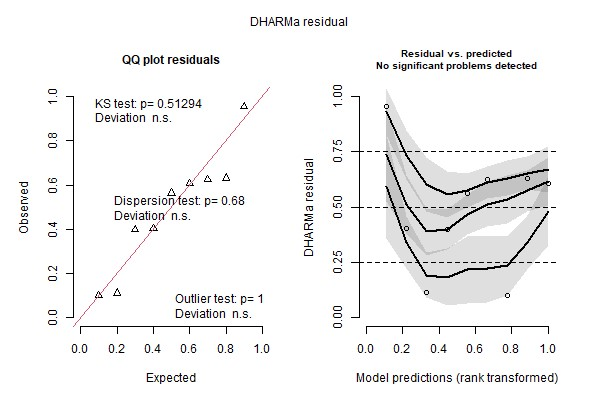 |  |
| March 20^th^ | ULC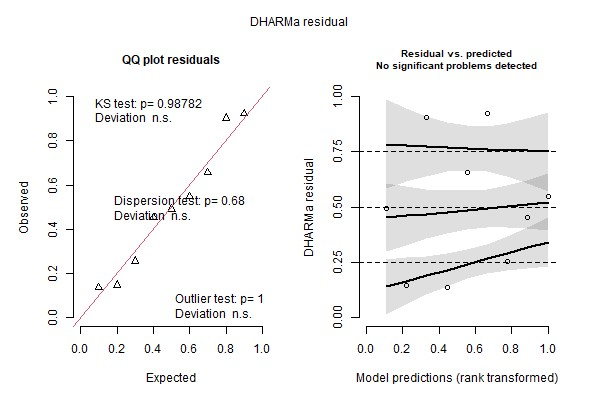 | UDC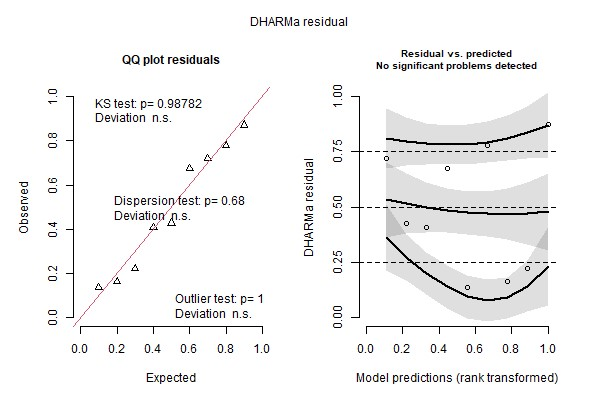 | LS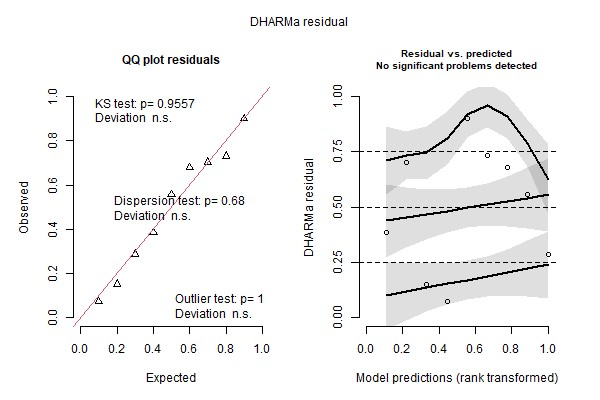 | DS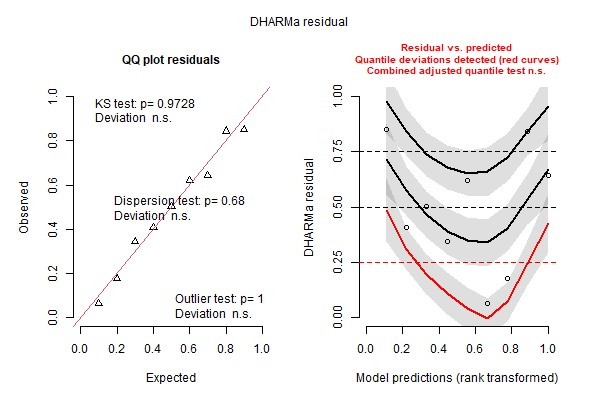 |
|  | M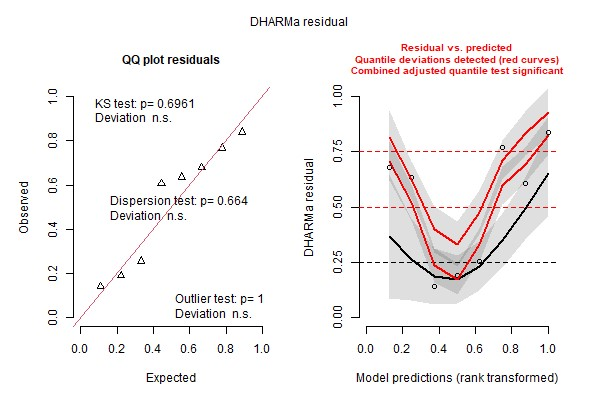 | L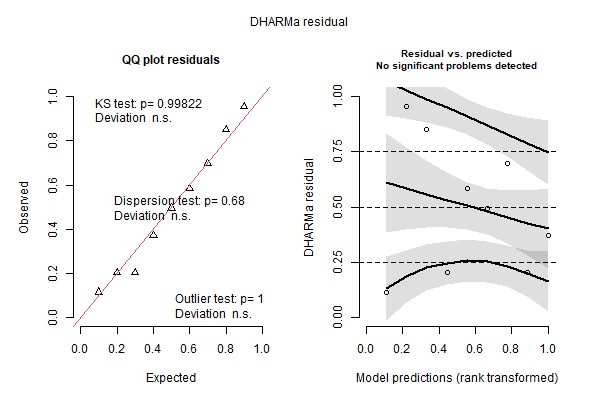 | O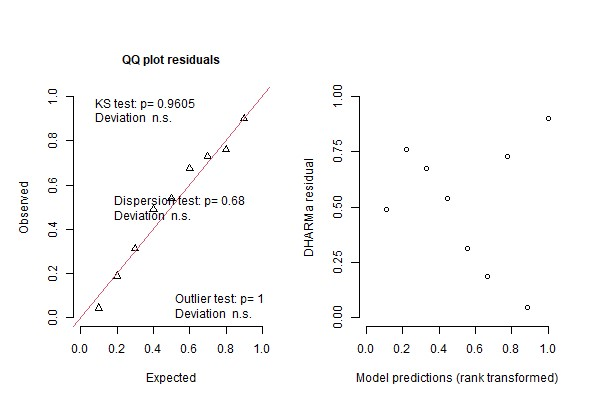 |  |
| March 26^th^ | ULC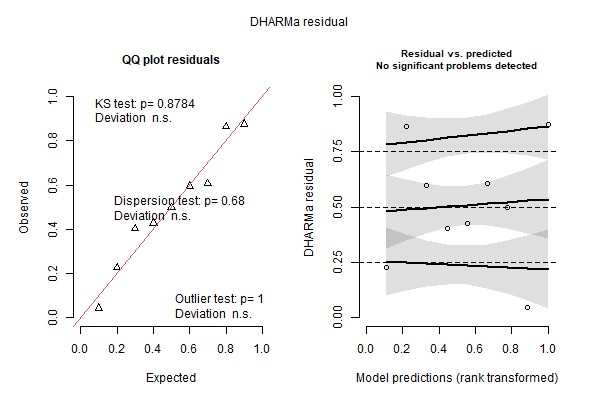 | UDC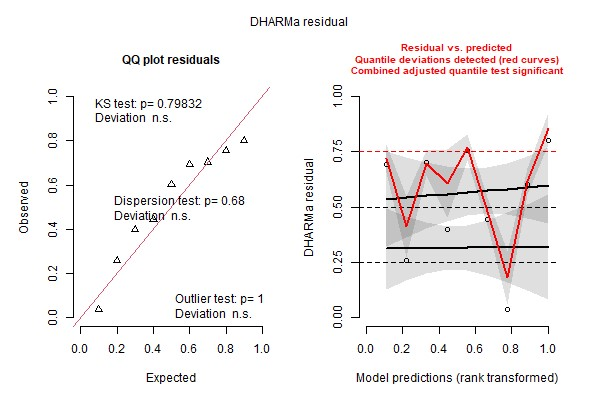 | LS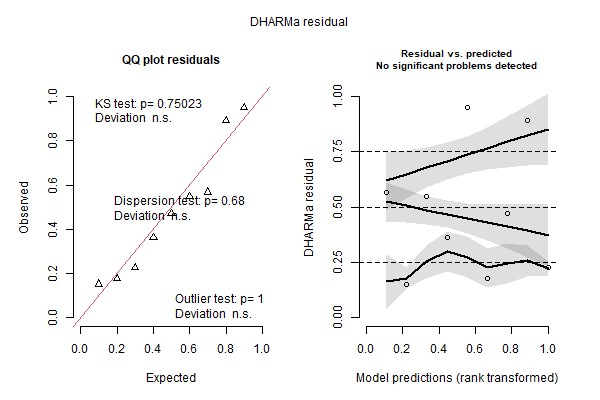 | DS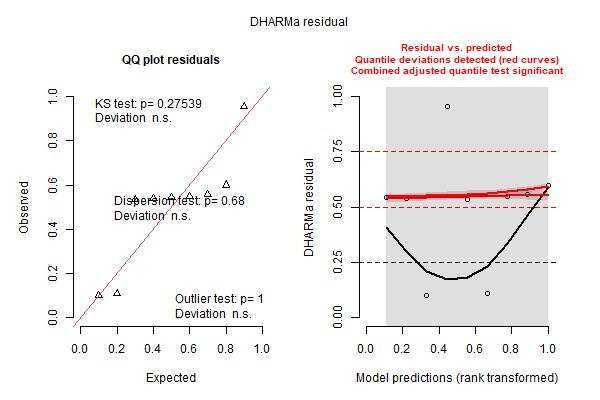 |
|  | M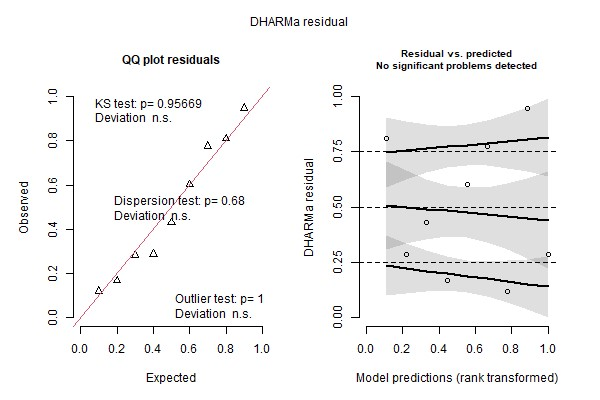 | L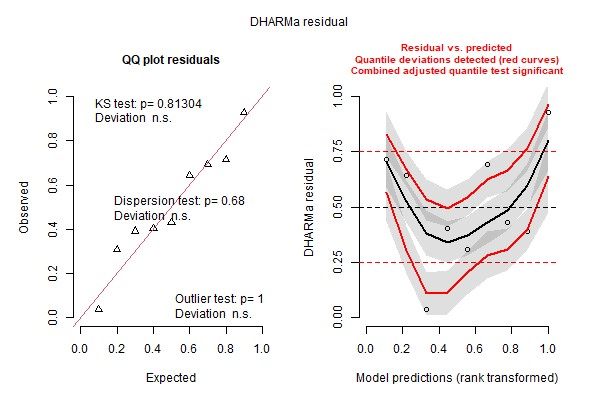 | O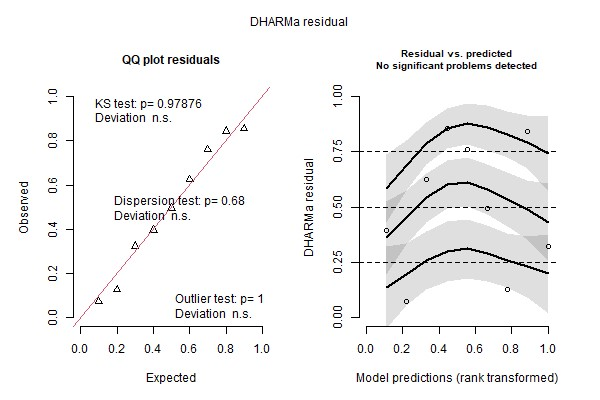 |  |
| April | ULC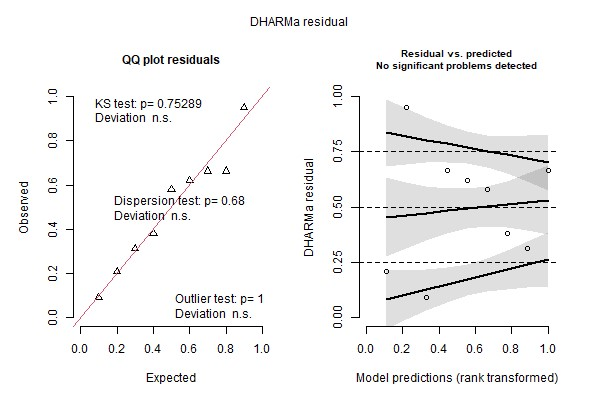 | UDC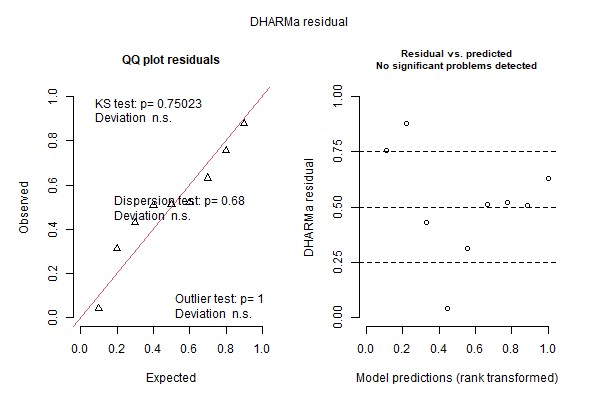 | LS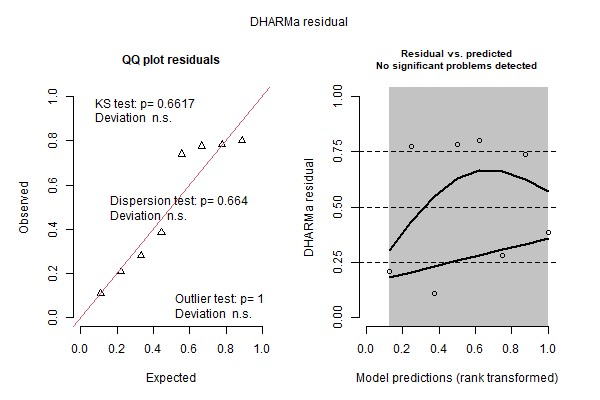 | DS  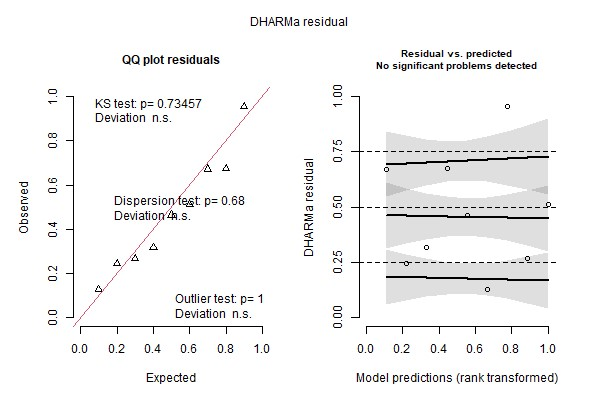 |
|  | M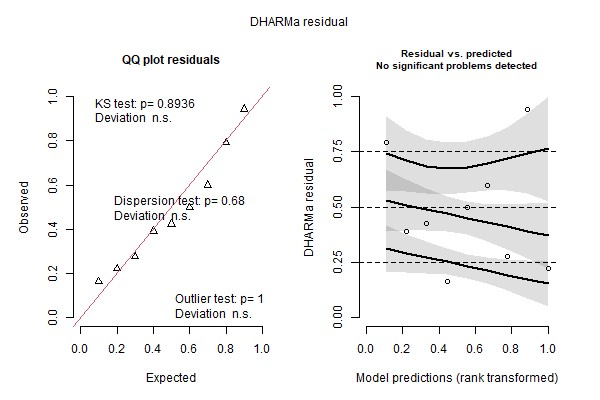 | L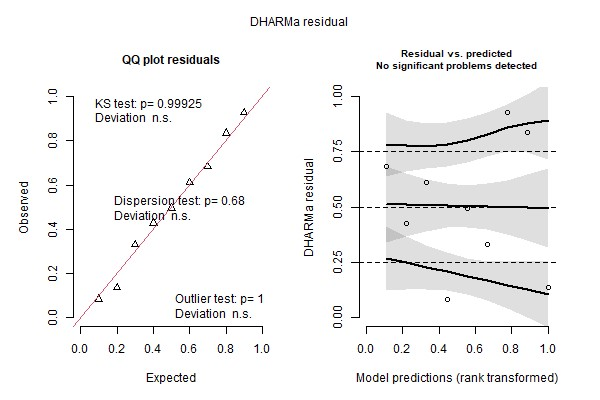 | O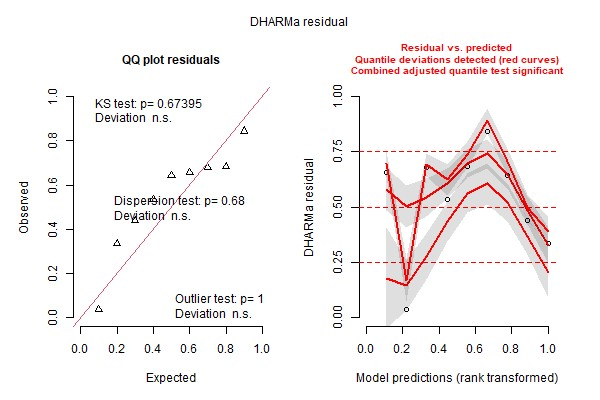 |  |
| May | ULC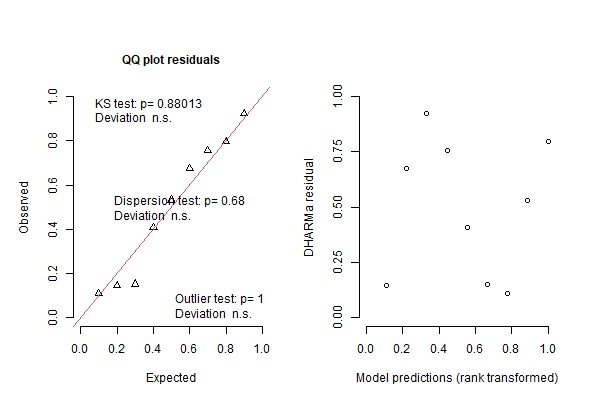 | UDC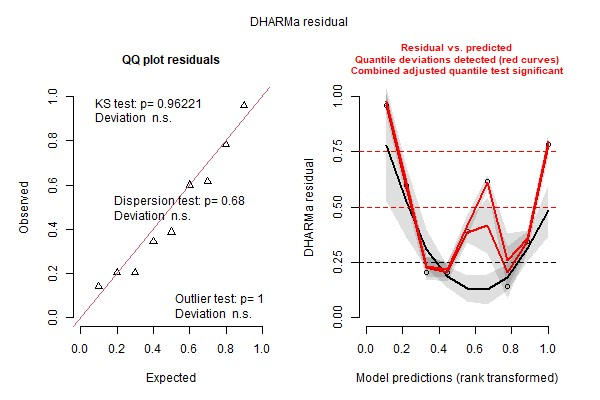 | LS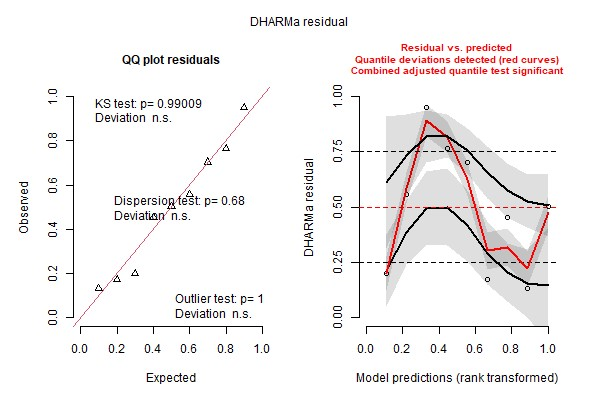 | DS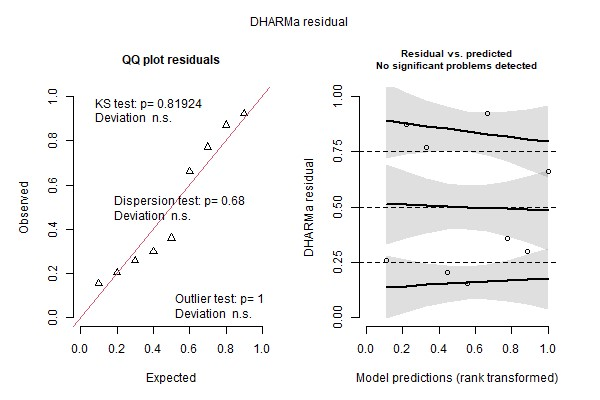 |
|  | M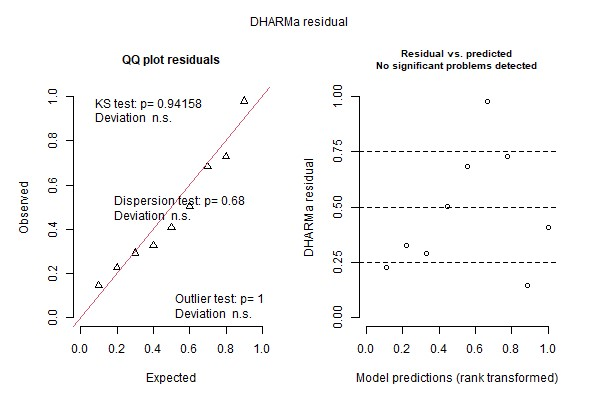 | L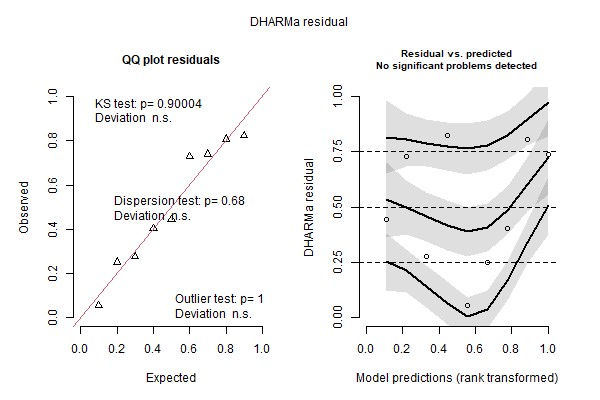 | O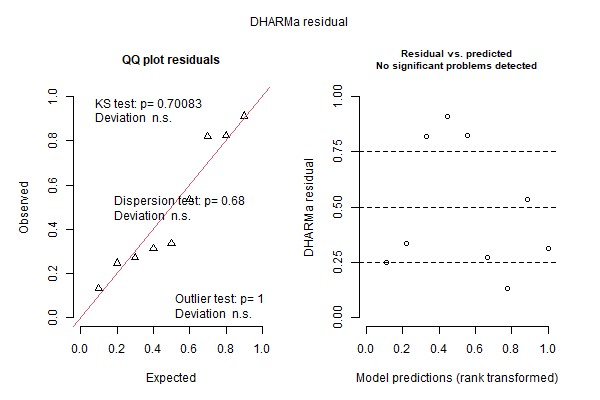 |  |
| June | ULC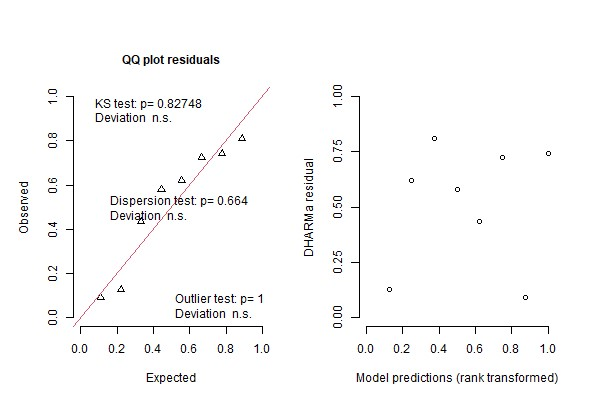 | UDC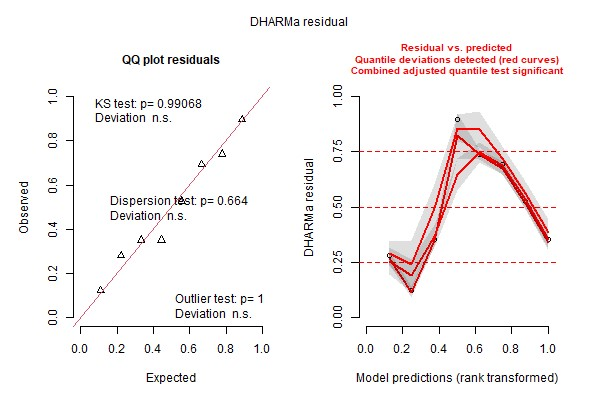 | LS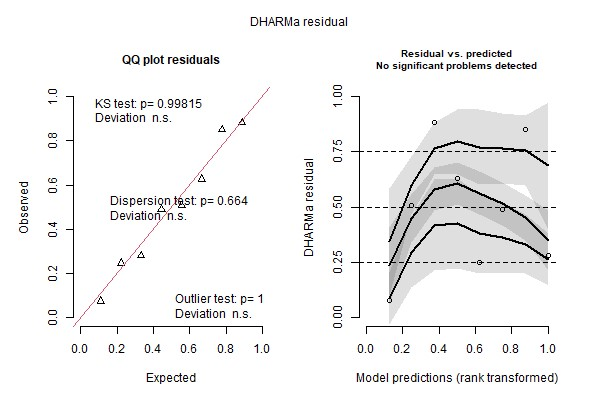 | DS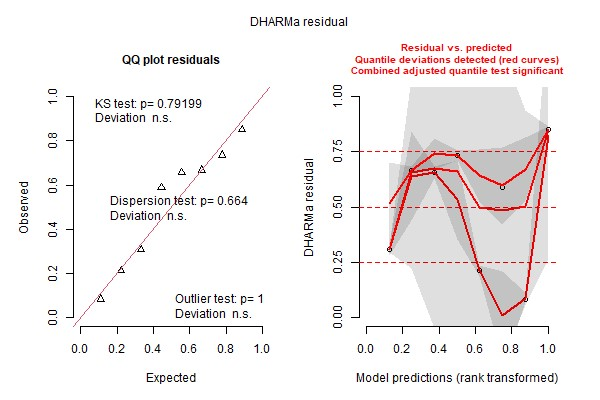 |
|  | M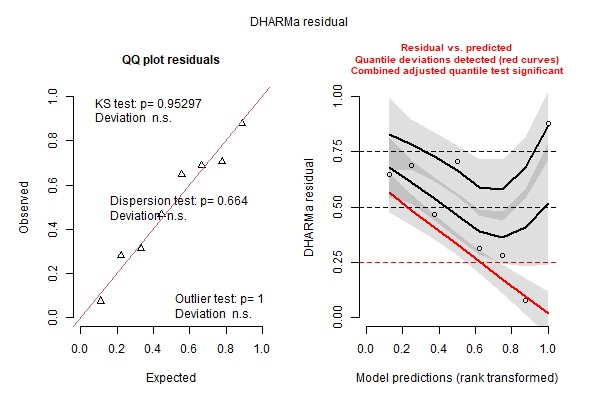 | L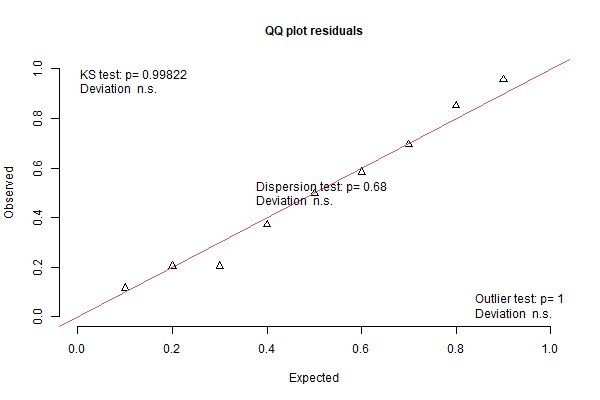 | O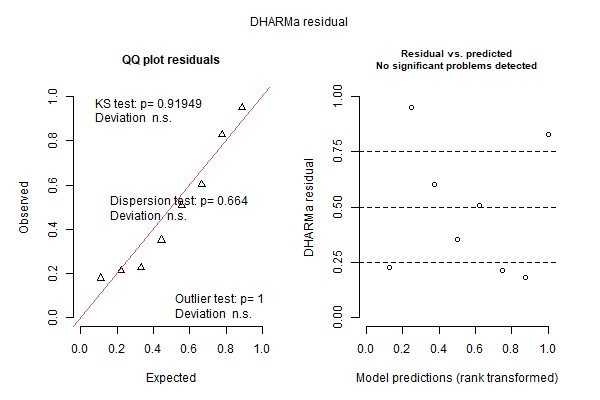 |  |
| August | ULC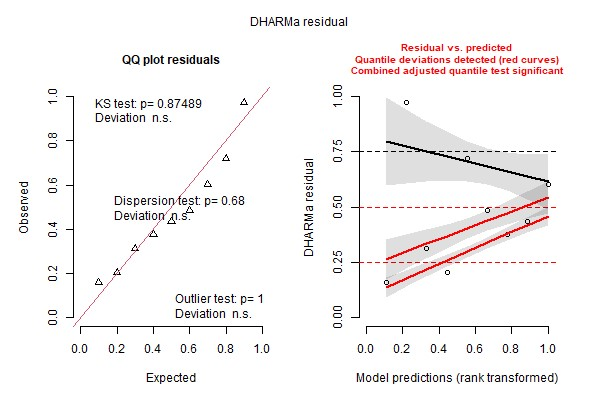 | UDC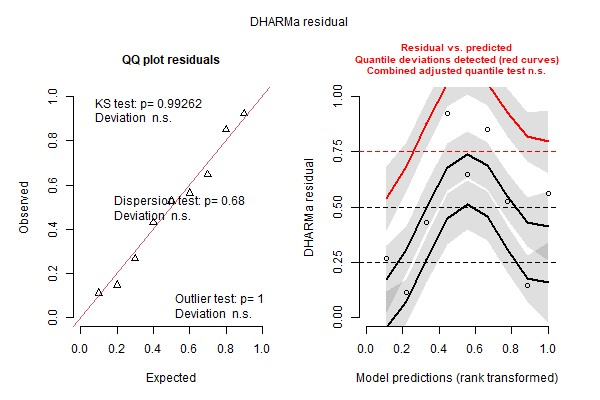 | LS  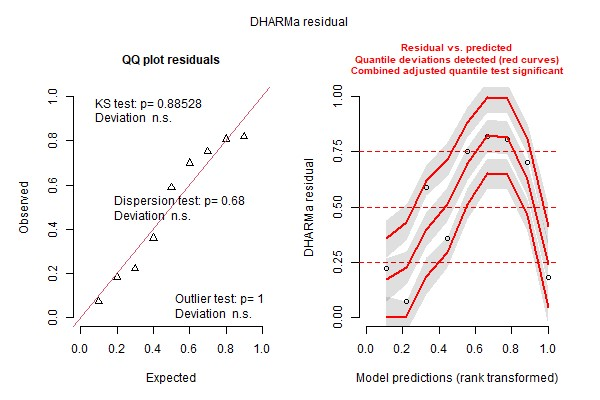 | DS  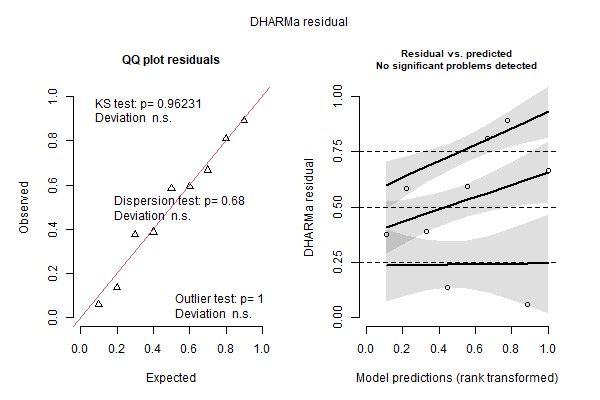 |
|  | M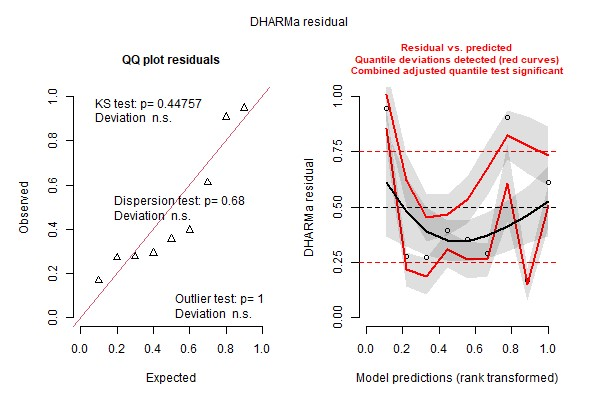 | L  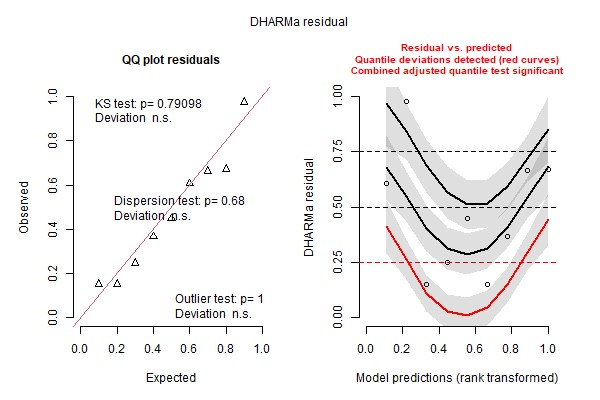 | O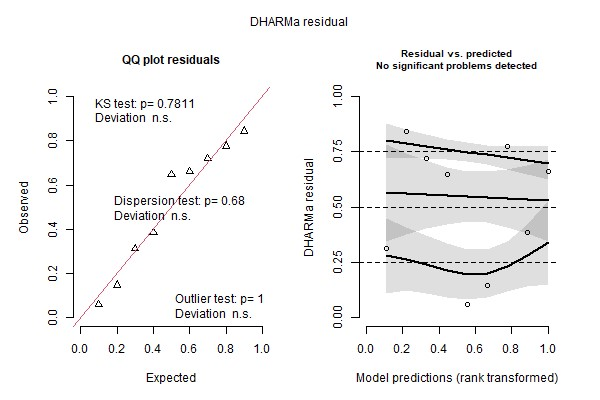 |  |

| Table S1: 2^nd^-order polynomial model summaries investigating whether there are diel patterns in FMC for each month, with additional sample days in March, and at each canopy layer. ULC = upper live canopy, UDC = upper dead canopy, LS = live stems, DS = dead stems, L = litter, M = moss, O = organic layer. | | | | | | |
| --- | --- | --- | --- | --- | --- | --- |
|  | *Estimate* | *Std. error* | *T-statistic* | *P-value (coefficients)* | *R2* | *P-value (test)* |
| March 19th | | | | | | |
| *ULC* | | | | | | |
| Intercept | 89.763 | 6.822 | 13.157 | 0.000 | 0.039 | 0.745 |
| Time | 2.347 | 3.133 | 0.749 | 0.465 |  |  |
| I(Time^2) | -0.237 | 0.306 | -0.775 | 0.451 |  |  |
| *UDC* |  |  |  |  |  |  |
| Intercept | 26.033 | 1.625 | 16.019 | 0.000 | 0.608 | 0.01 |
| Time | -2.614 | 0.746 | -3.503 | 0.003 |  |  |
| I(Time^2) | 0.195 | 0.073 | 2.681 | 0.017 |  |  |
| *LS* |  |  |  |  |  |  |
| Intercept | 81.575 | 3.865 | 21.106 | 0.000 | 0.059 | 0.636 |
| Time | 1.623 | 1.775 | 0.914 | 0.375 |  |  |
| I(Time^2) | -0.166 | 0.173 | -0.961 | 0.352 |  |  |
| *DS* |  |  |  |  |  |  |
| Intercept | 29.669 | 3.343 | 8.874 | 0.000 | 0.259 | 0.122 |
| Time | -3.205 | 1.541 | -2.080 | 0.056 |  |  |
| I(Time^2) | 0.278 | 0.149 | 1.857 | 0.084 |  |  |
| *L* |  |  |  |  |  |  |
| Intercept | 165.390 | 44.964 | 3.678 | 0.003 | 0.048 | 0.720 |
| Time | -15.981 | 20.640 | -0.774 | 0.453 |  |  |
| I(Time^2) | 1.416 | 2.031 | 0.697 | 0.498 |  |  |
| *M* |  |  |  |  |  |  |
| Intercept | 226.514 | 54.480 | 4.158 | 0.001 | 0.203 | 0.205 |
| Time | -36.712 | 25.613 | -1.433 | 0.174 |  |  |
| I(Time^2) | 4.159 | 2.503 | 1.662 | 0.119 |  |  |
| *O* |  |  |  |  |  |  |
| Intercept | 354.508 | 18.621 | 19.038 | 0.000 | 0.310 | 0.062 |
| Time | -16.215 | 8.550 | -1.897 | 0.077 |  |  |
| I(Time^2) | 1.216 | 0.834 | 1.459 | 0.165 |  |  |
| March 20th | |  |  |  |  |  |
| *ULC* |  |  |  |  |  |  |
| Intercept | 191.329 | 17.812 | 10.742 | 0.000 | 0.849 | 0.003 |
| Time | -15.031 | 2.605 | -5.770 | 0.001 |  |  |
| I(Time^2) | 0.529 | 0.093 | 5.705 | 0.001 |  |  |
| *UDC* |  |  |  |  |  |  |
| Intercept | 130.261 | 17.113 | 7.612 | 0.000 | 0.88 | 0.002 |
| Time | -16.388 | 2.503 | -6.547 | 0.001 |  |  |
| I(Time^2) | 0.590 | 0.089 | 6.616 | 0.001 |  |  |
| *LS* |  |  |  |  |  |  |
| Intercept | 111.591 | 22.447 | 4.971 | 0.003 | 0.194 | 0.524 |
| Time | -3.941 | 3.283 | -1.200 | 0.275 |  |  |
| I(Time^2) | 0.140 | 0.117 | 1.201 | 0.275 |  |  |
| *DS* |  |  |  |  |  |  |
| Intercept | 104.080 | 32.456 | 3.207 | 0.018 | 0.515 | 0.114 |
| Time | -11.983 | 4.747 | -2.524 | 0.045 |  |  |
| I(Time^2) | 0.424 | 0.169 | 2.509 | 0.046 |  |  |
| *L* |  |  |  |  |  |  |
| Intercept | 32.873 | 227.731 | 0.144 | 0.890 | 0.178 | 0.556 |
| Time | 19.682 | 33.309 | 0.591 | 0.576 |  |  |
| I(Time^2) | -0.791 | 1.186 | -0.667 | 0.529 |  |  |
| *M* |  |  |  |  |  |  |
| Intercept | -85.877 | 526.544 | -0.163 | 0.877 | 0.084 | 0.803 |
| Time | 27.082 | 77.678 | 0.349 | 0.742 |  |  |
| I(Time^2) | -0.842 | 2.777 | -0.303 | 0.774 |  |  |
| *O* |  |  |  |  |  |  |
| Intercept | 405.253 | 154.971 | 2.615 | 0.040 | 0.098 | 0.734 |
| Time | -10.859 | 22.667 | -0.479 | 0.649 |  |  |
| I(Time^2) | 0.343 | 0.807 | 0.425 | 0.686 |  |  |
| March 26th | |  |  |  |  |  |
| *ULC* |  |  |  |  |  |  |
| Intercept | 102.709 | 4.046 | 25.384 | 0.000 | 0.236 | 0.133 |
| Time | -3.738 | 1.858 | -2.012 | 0.063 |  |  |
| I(Time^2) | 0.325 | 0.181 | 1.792 | 0.093 |  |  |
| *UDC* |  |  |  |  |  |  |
| Intercept | 19.529 | 1.264 | 15.455 | 0.000 | 0.770 | 0.001 |
| Time | -3.407 | 0.580 | -5.871 | 0.000 |  |  |
| I(Time^2) | 0.373 | 0.057 | 6.599 | 0.000 |  |  |
| *LS* |  |  |  |  |  |  |
| Intercept | 82.536 | 3.200 | 25.792 | 0.000 | 0.047 | 0.696 |
| Time | -1.032 | 1.469 | -0.702 | 0.493 |  |  |
| I(Time^2) | 0.114 | 0.143 | 0.796 | 0.439 |  |  |
| *DS* |  |  |  |  |  |  |
| Intercept | 21.179 | 1.308 | 16.188 | 0.000 | 0.633 | 0.005 |
| Time | -3.020 | 0.601 | -5.027 | 0.000 |  |  |
| I(Time^2) | 0.297 | 0.059 | 5.078 | 0.000 |  |  |
| *L* |  |  |  |  |  |  |
| Intercept | 27.908 | 7.313 | 3.816 | 0.002 | 0.060 | 0.632 |
| Time | 2.198 | 3.358 | 0.655 | 0.523 |  |  |
| I(Time^2) | -0.157 | 0.327 | -0.479 | 0.639 |  |  |
| *M* |  |  |  |  |  |  |
| Intercept | 59.448 | 25.798 | 2.304 | 0.036 | 0.168 | 0.253 |
| Time | -11.926 | 11.845 | -1.007 | 0.330 |  |  |
| I(Time^2) | 1.496 | 1.155 | 1.295 | 0.215 |  |  |
| *O* |  |  |  |  |  |  |
| Intercept | 326.929 | 15.302 | 21.366 | 0.000 | 0.371 | 0.031 |
| Time | -16.430 | 7.026 | -2.338 | 0.034 |  |  |
| I(Time^2) | 1.284 | 0.685 | 1.874 | 0.081 |  |  |
| April |  |  |  |  |  |  |
| *ULC* |  |  |  |  |  |  |
| Intercept | 152.011 | 29.833 | 5.095 | 0.002 | 0.411 | 0.205 |
| Time | -8.869 | 4.363 | -2.032 | 0.088 |  |  |
| I(Time^2) | 0.317 | 0.155 | 2.044 | 0.087 |  |  |
| *UDC* |  |  |  |  |  |  |
| Intercept | 35.045 | 5.735 | 6.111 | 0.001 | 0.711 | 0.024 |
| Time | -3.009 | 0.839 | -3.587 | 0.012 |  |  |
| I(Time^2) | 0.103 | 0.030 | 3.465 | 0.013 |  |  |
| *LS* |  |  |  |  |  |  |
| Intercept | 94.730 | 22.015 | 4.303 | 0.008 | 0.069 | 0.837 |
| Time | -1.518 | 3.248 | -0.467 | 0.660 |  |  |
| I(Time^2) | 0.058 | 0.116 | 0.496 | 0.641 |  |  |
| *DS* |  |  |  |  |  |  |
| Intercept | 28.615 | 13.189 | 2.170 | 0.073 | 0.553 | 0.089 |
| Time | -1.550 | 1.929 | -0.803 | 0.452 |  |  |
| I(Time^2) | 0.041 | 0.069 | 0.591 | 0.576 |  |  |
| *L* |  |  |  |  |  |  |
| Intercept | 3.901 | 273.199 | 0.014 | 0.989 | 0.136 | 0.646 |
| Time | 6.553 | 39.959 | 0.164 | 0.875 |  |  |
| I(Time^2) | -0.123 | 1.422 | -0.086 | 0.934 |  |  |
| *M* |  |  |  |  |  |  |
| Intercept | 103.352 | 71.495 | 1.446 | 0.198 | 0.331 | 0.3 |
| Time | -11.299 | 10.457 | -1.080 | 0.321 |  |  |
| I(Time^2) | 0.361 | 0.372 | 0.969 | 0.370 |  |  |
| *O* |  |  |  |  |  |  |
| Intercept | 471.713 | 178.893 | 2.637 | 0.039 | 0.158 | 0.597 |
| Time | -27.462 | 26.165 | -1.050 | 0.334 |  |  |
| I(Time^2) | 0.963 | 0.931 | 1.034 | 0.341 |  |  |
| May |  |  |  |  |  |  |
| *ULC* |  |  |  |  |  |  |
| Intercept | 129.685 | 60.073 | 2.159 | 0.074 | 0.293 | 0.354 |
| Time | -2.173 | 8.787 | -0.247 | 0.813 |  |  |
| I(Time^2) | 0.038 | 0.313 | 0.121 | 0.908 |  |  |
| *UDC* |  |  |  |  |  |  |
| Intercept | 44.785 | 10.041 | 4.460 | 0.004 | 0.824 | 0.005 |
| Time | -4.685 | 1.469 | -3.190 | 0.019 |  |  |
| I(Time^2) | 0.148 | 0.052 | 2.839 | 0.030 |  |  |
| *LS* |  |  |  |  |  |  |
| Intercept | 75.082 | 46.317 | 1.621 | 0.156 | 0.085 | 0.765 |
| Time | 2.596 | 6.774 | 0.383 | 0.715 |  |  |
| I(Time^2) | -0.105 | 0.241 | -0.434 | 0.680 |  |  |
| *DS* |  |  |  |  |  |  |
| Intercept | 40.019 | 16.015 | 2.499 | 0.047 | 0.759 | 0.014 |
| Time | -3.300 | 2.342 | -1.409 | 0.209 |  |  |
| I(Time^2) | 0.089 | 0.083 | 1.072 | 0.325 |  |  |
| *L* |  |  |  |  |  |  |
| Intercept | -29.909 | 113.558 | -0.263 | 0.801 | 0.429 | 0.186 |
| Time | 12.687 | 16.609 | 0.764 | 0.474 |  |  |
| I(Time^2) | -0.545 | 0.591 | -0.921 | 0.393 |  |  |
| *M* |  |  |  |  |  |  |
| Intercept | 116.462 | 55.196 | 2.110 | 0.079 | 0.385 | 0.232 |
| Time | -14.914 | 8.073 | -1.847 | 0.114 |  |  |
| I(Time^2) | 0.516 | 0.287 | 1.794 | 0.123 |  |  |
| *O* |  |  |  |  |  |  |
| Intercept | 205.377 | 264.220 | 0.777 | 0.467 | 0.004 | 0.987 |
| Time | 6.126 | 38.646 | 0.159 | 0.879 |  |  |
| I(Time^2) | -0.213 | 1.376 | -0.155 | 0.882 |  |  |
| June |  |  |  |  |  |  |
| *ULC* |  |  |  |  |  |  |
| Intercept | 287.895 | 57.658 | 4.993 | 0.004 | 0.757 | 0.029 |
| Time | -19.768 | 8.093 | -2.443 | 0.058 |  |  |
| I(Time^2) | 0.619 | 0.278 | 2.223 | 0.077 |  |  |
| UDC |  |  |  |  |  |  |
| Intercept | 24.018 | 11.448 | 2.098 | 0.090 | 0.536 | 0.147 |
| Time | -2.240 | 1.607 | -1.394 | 0.222 |  |  |
| I(Time^2) | 0.084 | 0.055 | 1.525 | 0.188 |  |  |
| *LS* |  |  |  |  |  |  |
| Intercept | 193.070 | 40.129 | 4.811 | 0.005 | 0.706 | 0.047 |
| Time | -11.331 | 5.633 | -2.012 | 0.100 |  |  |
| I(Time^2) | 0.351 | 0.194 | 1.813 | 0.130 |  |  |
| DS |  |  |  |  |  |  |
| Intercept | 11.323 | 18.820 | 0.602 | 0.574 | 0.116 | 0.735 |
| Time | 0.238 | 2.642 | 0.090 | 0.932 |  |  |
| I(Time^2) | -0.013 | 0.091 | -0.145 | 0.890 |  |  |
| *L* |  |  |  |  |  |  |
| Intercept | -61.166 | 120.158 | -0.509 | 0.632 | 0.501 | 0.176 |
| Time | 14.753 | 16.866 | 0.875 | 0.422 |  |  |
| I(Time^2) | -0.589 | 0.580 | -1.015 | 0.357 |  |  |
| *M* |  |  |  |  |  |  |
| Intercept | 184.299 | 71.277 | 2.586 | 0.049 | 0.719 | 0.042 |
| Time | -26.164 | 10.005 | -2.615 | 0.047 |  |  |
| I(Time^2) | 0.956 | 0.344 | 2.776 | 0.039 |  |  |
| *O* |  |  |  |  |  |  |
| Intercept | -98.260 | 352.025 | -0.279 | 0.791 | 0.203 | 0.567 |
| Time | 44.024 | 49.413 | 0.891 | 0.414 |  |  |
| I(Time^2) | -1.430 | 1.700 | -0.841 | 0.439 |  |  |
| August |  |  |  |  |  |  |
| *ULC* |  |  |  |  |  |  |
| Intercept | 290.15 | 33.39 | 8.69 | 0.00 | 0.859 | 0.003 |
| Time | -23.51 | 4.88 | -4.81 | 0.00 |  |  |
| I(Time^2) | 0.78 | 0.17 | 4.50 | 0.00 |  |  |
| *UDC* |  |  |  |  |  |  |
| Intercept | 74.343 | 9.165 | 8.112 | 0.000 | 0.896 | 0.001 |
| Time | -8.631 | 1.340 | -6.439 | 0.001 |  |  |
| I(Time^2) | 0.294 | 0.048 | 6.161 | 0.001 |  |  |
| *LS* |  |  |  |  |  |  |
| Intercept | 154.192 | 40.683 | 3.790 | 0.009 | 0.382 | 0.236 |
| Time | -8.112 | 5.950 | -1.363 | 0.222 |  |  |
| I(Time^2) | 0.265 | 0.212 | 1.249 | 0.258 |  |  |
| *DS* |  |  |  |  |  |  |
| Intercept | 73.005 | 13.872 | 5.263 | 0.002 | 0.861 | 0.003 |
| Time | -7.477 | 2.029 | -3.685 | 0.010 |  |  |
| I(Time^2) | 0.237 | 0.072 | 3.281 | 0.017 |  |  |
| *L* |  |  |  |  |  |  |
| Intercept | 529.501 | 80.733 | 6.559 | 0.001 | 0.862 | 0.003 |
| Time | -68.582 | 11.808 | -5.808 | 0.001 |  |  |
| I(Time^2) | 2.367 | 0.420 | 5.630 | 0.001 |  |  |
| *M* |  |  |  |  |  |  |
| Intercept | 594.415 | 317.899 | 1.870 | 0.111 | 0.654 | 0.042 |
| Time | -61.114 | 46.497 | -1.314 | 0.237 |  |  |
| I(Time^2) | 1.755 | 1.655 | 1.060 | 0.330 |  |  |
| *O* |  |  |  |  |  |  |
| Intercept | 344.908 | 191.059 | 1.805 | 0.121 | 0.19 | 0.531 |
| Time | -31.210 | 27.945 | -1.117 | 0.307 |  |  |
| I(Time^2) | 1.075 | 0.995 | 1.081 | 0.321 |  |  |
|  |  |  |  |  |  |  |

| Table S2: Range in temperature and humidity (max – min) for each sample day. | | | | | | | |
| --- | --- | --- | --- | --- | --- | --- | --- |
|  | **March 19th** | **March 20th** | **March 26th** | **April** | **May** | **June** | **August** |
| Temperature (°C) | 4.11 | 4.11 | 4.72 | 3.28 | 5.38 | 2.56 | 4.27 |
| Humidity (%) | 36 | 29 | 13 | 7 | 17 | 10 | 50 |

| Table S3: Mean FMC for each sample day. Numbers in bold indicate that mean FMC is lower than recorded ignition thresholds recorded by Santana and Mars [1] and Grau-Andrés et al. [2]. Red text indicates that daily mean FMC value lies above the ignition threshold, but at points during the sample day FMC fell below the ignition threshold. | | | | | | | |
| --- | --- | --- | --- | --- | --- | --- | --- |
| Sample | March 19th | March 20th | March 26th | April | May | June | August |
| ULC | 94.0 | 88.1 | 94.3 | 92.2 | 106.9 | 134.6 | 119.6 |
| LS | 84.4 | 84.9 | 81.0 | 85.2 | 90.2 | 104.5 | 94.3 |
| UDC | 19.1 | 20.3 | 14.3 | 13.9 | 9.3 | 9.7 | 13.1 |
| DS | 22.5 | 22.2 | 15.5 | 15.1 | 11.9 | 11.9 | 16.4 |
| L | 130.3 | 148.0 | **33.9** | 70.8 | **37.3** | **25.9** | **49.0** |
| M | 176.3 | 122.6 | **47.2** | **18.3** | **12.1** | **10.9** | **94.5** |
| O | 312.0 | 322.7 | 285.4 | 282.3 | 247.9 | 232.0 | **125.8** |

References

1. Santana, V.M. and Marrs, R.H., 2014. Flammability properties of British heathland and moorland vegetation: models for predicting fire ignition. Journal of environmental management, 139, pp.88-96.

2. Grau-Andrés, R., Davies, G.M., Gray, A., Scott, E.M. and Waldron, S., 2018. Fire severity is more sensitive to low fuel moisture content on Calluna heathlands than on peat bogs. Science of the total environment, 616, pp.1261-1269.
